# Effect of Nano-AlN Particles on the Microstructure and Mechanical Properties of Mg-Al-Nd-Mn Alloy

**DOI:** 10.3390/ma18174104

**Published:** 2025-09-01

**Authors:** Xiang Zhang, Kun Zhang, Yuyang Gao, Ang Zhang, Jing Zhao, Yuanlin Li, Zhihua Dong, Yan Song, Tian Li, Bin Jiang

**Affiliations:** 1 National Engineering Research Center for Magnesium Alloys, College of Materials Science and Engineering, Chongqing University, Chongqing 400044, China; 202309021116t@stu.cqu.edu.cn (X.Z.); 18723616129@163.com (K.Z.); 202209021048@stu.cqu.edu.cn (Y.L.); dzhihua@cqu.edu.cn (Z.D.); jiangbinrong@cqu.edu.cn (B.J.); 2 National Key Laboratory of Advanced Casting Technologies, Chongqing University, Chongqing 400044, China; 3 Rare Earth Advanced Materials Technology Innovation Center, Inner Mongolia Northern Rare Earth Advanced Materials Technology Innovation Co., Ltd., Baotou 014030, China; zhaojing@reamtic.cn; 4 Department of Components and Materials Test & Evaluation Research Center, China Automotive Engineering Research Institute (CAERI), Chongqing 401122, China; 99993170@caeri.com.cn; 5 Zhejiang Wanfeng Precision Manufacturing Co., Ltd., Shaoxing 312400, China; l0101270@gmail.com

**Keywords:** Mg-4Al-2Nd-0.3Mn alloys, nano-AlN particles, high-temperature mechanical properties

## Abstract

The addition of AlN particles effectively refined the α-Mg grain size of Mg-4Al-2Nd-0.3Mn (AE42) alloy, as well as the size of acicular Al11Nd3 and blocky Al2Nd phases, while promoting the precipitation of the Al8Mn5 phase. The 2.0 wt.% AlN/AE42 composite exhibited optimal room- and high-temperature mechanical properties. At room temperature, the ultimate tensile strength (UTS), yield strength (YS), and fracture strain (ε) of AE42 alloy and 2.0 wt.% AlN/AE42 composite were 191 MPa, 86 MPa, 12.1% and 219 MPa, 107 MPa, and 13.8%, respectively. At 150 °C, 200 °C, and 250 °C, the UTS values of the 2.0 wt.% AlN/AE42 composite were 178 MPa, 152 MPa, and 139 MPa, respectively. At high temperatures, AlN particles synergistically enhanced the strength and plasticity of the composite by suppressing grain boundary sliding and promoting twinning.

## 1. Introduction

Magnesium (Mg) alloys, as the lightest metallic structural materials in current industrial applications, were attracting increasing attention in modern industries [1,2,3]. Driven by energy conservation and emission reduction demands, the application of Mg alloys was no longer limited to non-heat-resistant automotive components. Many critical structural parts imposed higher requirements on their high-temperature mechanical properties [4,5]. The mechanical properties of the currently commercially used Mg-Al-Zn or Mg-Al-Mn alloys sharply declined when the temperature exceeded 120 °C. The reason was that the Mg_17_Al_12_ phase with poor thermal stability was prone to soften or coarsen at temperatures above 120 °C, resulting in diminished effectiveness in impeding dislocation motion and consequently reduced high-temperature mechanical performance [6]. Adding rare earth (RE) elements (e.g., Nd) enhanced the heat resistance of magnesium alloys. Since Nd exhibited higher chemical affinity with Al than with Mg, it suppressed the formation of the Mg_17_Al_12_ phase [7,8].

The incorporation of reinforcement particles such as SiC, TiC, TiB_2_, and AlN into Mg alloys to form Mg matrix composites also enhanced their elevated-temperature mechanical properties [9,10,11,12]. Among these, AlN particles not only possessed advantages including high hardness, high melting point, low thermal expansion coefficient, and high stability, but also had a relatively low density of 3.2 g/cm^3^, meeting the lightweight requirements for structural components [13]. Furthermore, AlN particles had the same hexagonal close-packed (HCP) crystal structure as Mg with similar lattice parameters, enabling them to serve as nucleation sites for α-Mg grains. This significantly refined the alloy grains and thereby improved the alloy properties [14,15]. Additionally, with a melting point as high as 2200 °C, AlN particles themselves exhibited excellent heat-resistant characteristics. Their addition as reinforcements improved the heat resistance of alloys through Orowan strengthening, load transfer strengthening, and thermal mismatch strengthening effects [16]. Cao et al. [17] added 1 wt.% AlN particles into AZ91D alloy via ultrasonic-assisted stir casting, which increased the tensile strength at 200 °C from 135 MPa to 170 MPa. Lydia et al. [18] introduced 1 wt.% AlN particles into Mg-2.8Nd-1.2Gd-0.4Zr-0.3Zn alloy through ultrasonic stirring, significantly reducing both creep deformation and creep rate at 240 °C, thus enhancing the alloy’s creep resistance. Therefore, conducting research on AlN particle-reinforced Mg matrix composites is expected to further enhance their room- and high-temperature strength and plasticity.

In order to avoid agglomeration of the reinforcing phase and surface contamination caused by ex situ particles, 1.0, 2.0, and 3.0 wt.% AlN/AE42 composites were fabricated by the master alloy and casting method, as described in Ref. [19]. The effects of nano-sized AlN particles on the microstructure and mechanical properties of AE42 alloy at room temperature and high temperatures (150 °C, 200 °C, 250 °C) were investigated, and the influence law of nano-sized AlN particle content was explored, providing theoretical guidance for the design and development of high-strength and high-temperature-resistant Mg-Al-RE-based composites.

## 2. Materials and Methods

Using pure Mg (99.98 wt.%), pure aluminum (Al) (99.98 wt.%), Mg-30Nd (wt.%), Mg-5Mn (wt.%). and AlN/Mg master alloys as raw materials, AE42 alloy and 1.0, 2.0, 3.0 wt.% AlN/AE42 composites were prepared in a resistance furnace. The procedure commenced by preheating the resistance furnace to 500 °C, followed by placement of a steel crucible containing pure Mg and pure Al. The temperature was elevated to 720 °C under continuous CO_2_/SF_6_ (99:1) protective gas flow. Upon complete melting of the base metals, Mg-30Nd and Mg-5Mn master alloys were sequentially introduced into the molten bath. Subsequently, preheated AlN/Mg master alloy was added and dissolved. The melt then underwent 10 min dispersion treatment combining mechanical stirring with ultrasonic processing to homogenize AlN particles. Finally, after stirring was completed, surface dross was removed, and the melt was held isothermally for 10 min. Subsequently, the melt was poured into a counter-gravity metallic mold preheated to 200 °C. Following solidification, cast ingots (φ80 mm × 120 mm) of AlN/AE42 composites were obtained.

**Figure 1:**
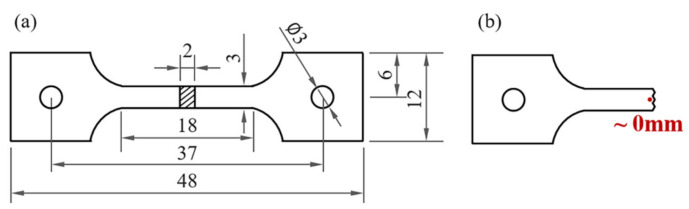
(**a**) Tensile specimen diagrams (room and high temperature; mm) [19], (**b**) observation positions of SEM and EBSD after breaking.

AlN/AE42 composites were identified by X-ray diffraction (XRD) (Ultima IV, Rigaku, Tokyo, Japan) with a scanning range of 10–90° at 4°/min. Microstructural characterization was performed using scanning electron microscopy (SEM) (JEOL JSM-7800F, Tokyo, Japan) (acceleration voltage: 15 kV) and transmission electron microscopy (TEM) (FEI Talos F200X, Thermo Fisher Scientific, Waltham, MA, USA). Grain size and twinning analysis were conducted via electron backscatter diffraction (EBSD) (scan step size: 2.5 μm). Tensile tests at a strain rate of 1 × 10^−3^ s^−1^ (tensile rate: 1.08 mm/min [20,21]) were carried out on a universal testing machine (CMT5105-100 kN, SUST, Shenzhen, China) equipped with a GX-1200A temperature control system for both room temperature (25 °C) and high temperatures (150 °C, 200 °C, and 250 °C). The samples used for the tensile test were prepared in accordance with the national standards of the People’s Republic of China (GB/T228.1-2021 and GB/T228.2-2015) [20,21], as illustrated in Figure 1a. Observation positions of SEM and EBSD after breaking are shown in Figure 1b. Prior to high-temperature testing, specimens were held isothermally for 10 min at target temperatures. For statistical reliability, a mechanical extensometer (CBY112-2.5, Changchun Institute of Mechanical Science, Changchun, China) was used, and three tests were performed per condition.

## 3. Results

### 3.1. Microstructural Evolution

**Figure 2:**
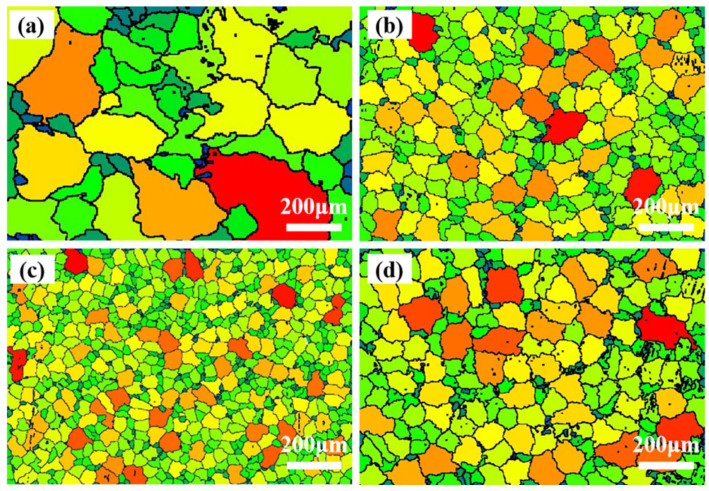
Grain size diagram of AlN/AE42 composites: (**a**) AE42, (**b**) 1.0 wt.% AlN/AE42, (**c**) 2.0 wt.% AlN/AE42, (**d**) 3.0 wt.% AlN/AE42.

AE42 alloy exhibited considerable variation in the sizes of α-Mg grain (34.6–489.2 μm), as shown in Figure 2a. Conversely, the size differences between the α-Mg grains in the composites are relatively small (Figure 2b–d). The α-Mg grain size of the composites was significantly smaller than that of the AE42 alloy, and it also showed a trend of first decreasing and then increasing with the increase in AlN content. The statistical average grain sizes of AE42 alloy, 1.0 wt.%, 2.0 wt.%, and 3.0 wt.% AlN/AE42 composites were 116.0 μm, 53.2 μm, 36.0 μm, and 50.6 μm, respectively. The refinement of the microstructure of AE42 alloy by AlN particles was attributed to the combined effect of the heterogeneous nucleation of the α-Mg phase on the surface of AlN particles and the obstruction of the growth of the primary α-Mg phase by AlN particles. In the 3.0 wt.% AlN/AE42 composite, coarsening of α-Mg grains was observed (Figure 2d).

**Figure 3:**
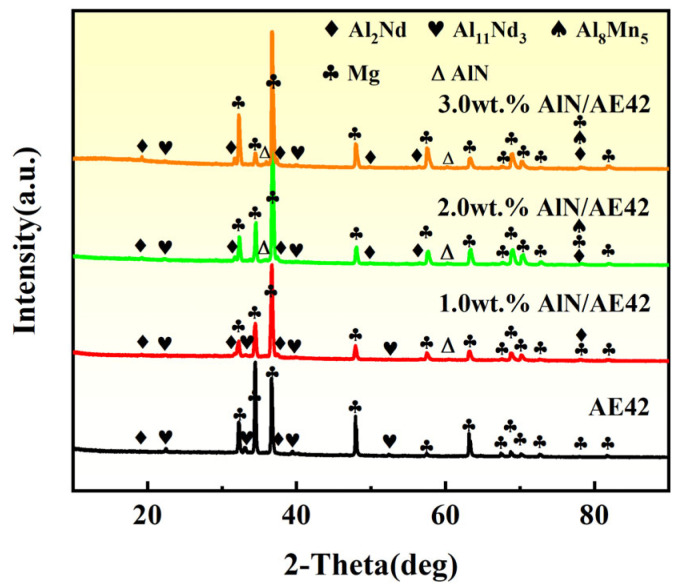
XRD pattern of AlN/AE42 composites with different AlN contents.

Figure 3 presents the XRD analysis results of AE42 alloy and its composites. The AE42 alloy primarily consisted of α-Mg, Al_2_Nd, and Al_11_Nd_3_ phases. Diffraction peaks of AlN phase were observed in 2.0 and 3.0 wt.% AlN/AE42 composites. Meanwhile, with the increase in the mass fraction of AlN particles, the diffraction peak intensity of the Al_2_Nd phase in the composite gradually increased. According to the research results of Hou et al. [22], the Al_2_Nd phase could serve as a heterogeneous nucleation site for α-Mg grains, further promoting the refinement of α-Mg grains in composites. Additionally, since AlN/Mg master alloy was used as the carrier medium for AlN particles, the introduction of AlN into the AE42 alloy simultaneously incorporated extra Al through the melting of the Al_3_Mg_2_ phase [19], promoting the formation of the Al_8_Mn_5_ phase. Therefore, diffraction peaks of the Al_8_Mn_5_ phase were observed in the 3.0 wt.% AlN/AE42 composite material.

**Figure 4:**
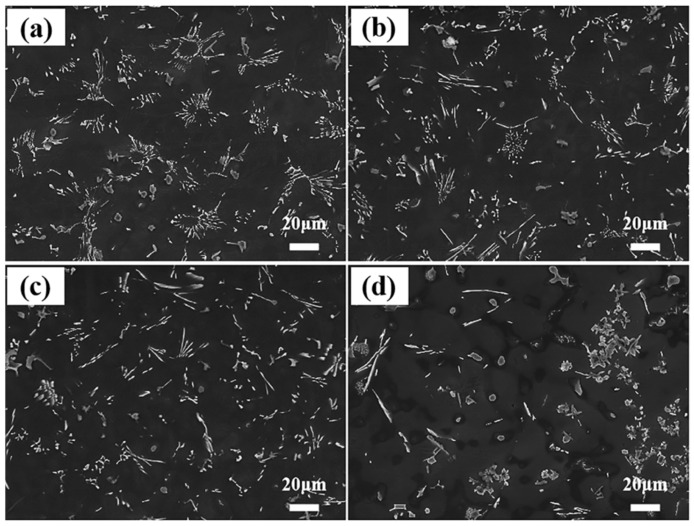
Microstructure micrographs of AlN/AE42 composites: (**a**) AE42, (**b**) 1.0 wt.% AlN/AE42, (**c**) 2.0 wt.% AlN/AE42, (**d**) 3.0 wt.% AlN/AE42.

The microstructures of AE42 alloy and its composites are presented in Figure 4. As shown in Figure 4a, the second phases in the AE42 alloy primarily exhibit a petal-like distribution of acicular phases and blocky phases. As presented in Figure 4b–d, after adding different contents of AlN particles, the morphology of second phases in the composites underwent noticeable changes: blocky phases within the petal-like structures decreased while short rod-shaped phases increased. Compared to the matrix alloy, the addition of AlN particles reduced the number of petal-like structures, making the distribution of Al_x_Nd_y_ second phases more dispersed and uniform. When the AlN content reached 3.0 wt.% (Figure 4d), the acicular phase significantly grew, and large areas of blocky and granular phase aggregated regions appeared.

**Figure 5:**
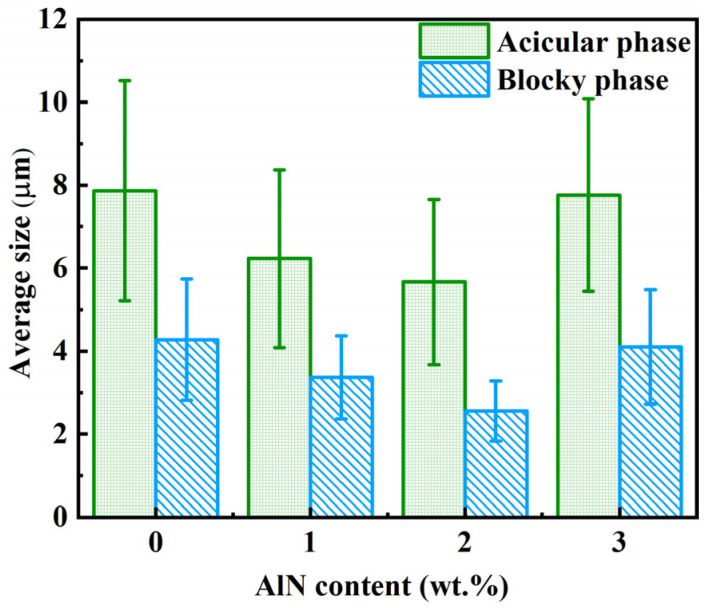
Statistical diagram of the second phase size of AlN/AE42 composites with different AlN contents.

Figure 5 presents the statistical results of second phase sizes in AE42 alloy and AlN/AE42 composites. The Al_x_Nd_y_ phases in the composites exhibited significantly smaller dimensions than those in the matrix alloy. With increasing AlN particle content, the size of Al_x_Nd_y_ phases first decreased then increased (based on 500 measurements). The statistically averaged sizes of acicular second phases were 7.9 μm, 6.2 μm, 5.7 μm, and 7.1 μm for AE42 alloy, 1.0 wt.%, 2.0 wt.%, and 3.0 wt.% AlN/AE42 composites, respectively. Corresponding sizes of blocky second phases measured 4.3 μm, 3.4 μm, 2.6 μm, and 3.9 μm. The higher the mass fraction and the better the dispersion of AlN particles, the stronger the effect on the refinement. Therefore, the sizes of the acicular and blocky Al_x_Nd_y_ second phases and the α-Mg grains reached their minimum values in the 2.0 wt.% AlN/AE42 composite, which were 5.7 μm, 2.6 μm, and 36.0 μm, respectively.

**Figure 6:**
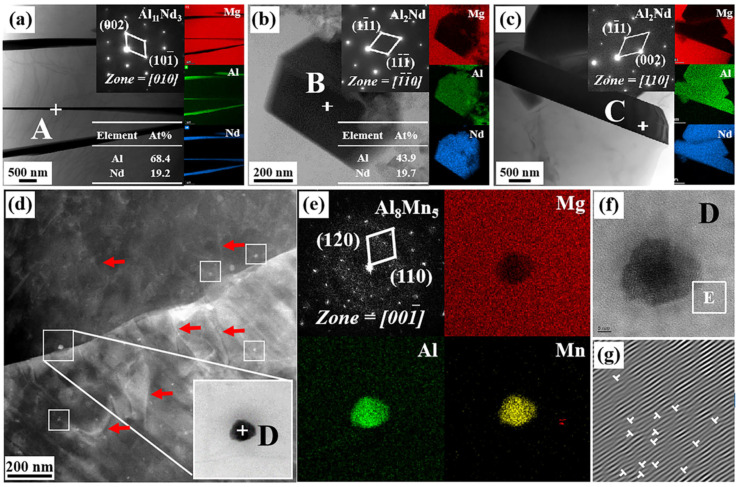
2.0 wt.% AlN/AE42 composites: (**a**) the TEM characterizations of Al_11_Nd_3_, (**b**,**c**) the TEM characterizations of Al_2_Nd, (**d**) the TEM characterizations of Al_8_Mn_5_ phase, (**e**) the SAED micrograph and EDS mapping of point D, (**f**) high-resolution TEM images of Al_8_Mn_5_ phase, (**g**) the IFFT micrograph of region E.

To further identify second phase morphologies and compositions in AE42 alloy and AlN/AE42 composites, TEM observations are conducted as shown in Figure 6. According to EDS point analysis (Figure 6a), the Al:Nd atomic ratio of acicular phase A is 3.56:1, closely matching the stoichiometric ratio of Al_11_Nd_3_ (11:3). Combined with selected area electron diffraction (SAED), phase A was identified as orthorhombic Al_11_Nd_3_ (a = 0.4359 nm, b = 1.2924 nm, c = 1.0017 nm [23]). The blocky phase B exhibits an Al:Nd atomic ratio of 2.22:1 (Figure 6b), closely matching the stoichiometric ratio of Al_2_Nd (2:1). This phase was further identified as face-centered cubic Al_2_Nd (a = 0.8000 nm [23]) through SAED analysis. Rod-shaped phase C was determined as Al_2_Nd based on SAED and elemental mapping. Hou et al. [22] reported identical short rod-shaped Al_2_Nd phases in Mg-Al-Nd alloys. Due to the introduction of the Al element in the composite, the formation of the Al_2_Nd phase was promoted, and its morphology changed from a blocky structure to a short rod-shaped structure. This was consistent with the XRD results (Figure 3) and the SEM image (Figure 4). A large number of dispersed nano-sized granular second phases (with an average size of 21.3 nm) were observed at the α-Mg grain boundaries and within the grains of the 2.0 wt.% AlN/AE42 composite, as shown in Figure 6d. SAED and EDS mapping (Figure 6e) confirmed these as the Al_8_Mn_5_ phase [24]. Al introduced via AlN addition promoted precipitation of nano-Al_8_Mn_5_ particles. Interactions between Al_8_Mn_5_ nanoparticles (white squares) and dislocations (red arrows) are shown in Figure 6d. According to the inverse Fourier transform result (Figure 6g), a high density of dislocations existed around Al_8_Mn_5_ particles, which helped to increase the YS of the 2.0 wt.% AlN/AE42 composite.

**Figure 7:**
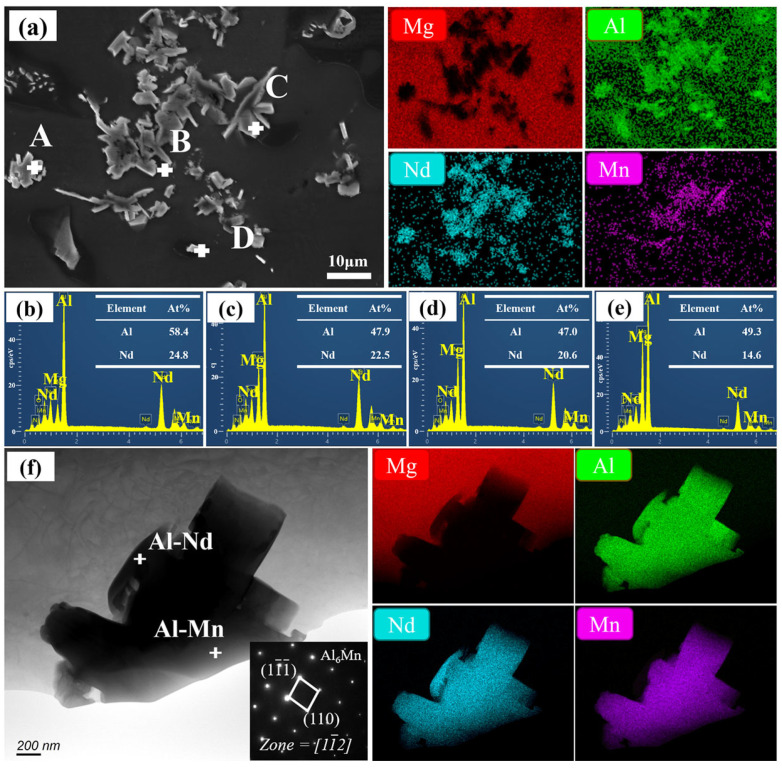
The second phase in 3.0 wt.% AlN/AE42: (**a**) the SEM micrograph and EDS mapping, (**b**) EDS results at point A, (**c**) EDS results at point B, (**d**) EDS results at point C, (**e**) EDS results at point D, (**f**) TEM micrograph and EDS mapping of the second phase of agglomeration.

Due to observed agglomeration regions in the 3.0 wt.% AlN/AE42 composite’s SEM image (Figure 4d), this area is characterized with results presented in Figure 7. Based on EDS point analysis results, points A, B, and C were identified as the Al_2_Nd phase, while point D corresponded to the Al_11_Nd_3_ phase. This indicated that the agglomerated region was mainly composed of the coarse Al_2_Nd and Al_11_Nd_3_ phases. EDS elemental mapping further revealed the presence of Al-Mn phases within this region. However, due to their small dimensions and close intergrowth with Al_x_Nd_y_ phases, these Al-Mn phases were poorly distinguishable. Further TEM investigation (Figure 7f) uncovered similar structures in the 3.0 wt.% AlN/AE42 composite. Through SAED and mapping images, the Al-Mn phase was confirmed as Al_6_Mn [25]. Compared to the Al_8_Mn_5_ phase observed in the 2.0 wt.% composite, the Al_6_Mn phase exhibited a significantly larger size. It was speculated that the change in Al-Mn phase composition was due to the increase in AlN content, and the subsequent introduction of more Al elements, thereby forming the Al_6_Mn second phase with its high Al:Mn atomic ratio.

**Figure 8:**
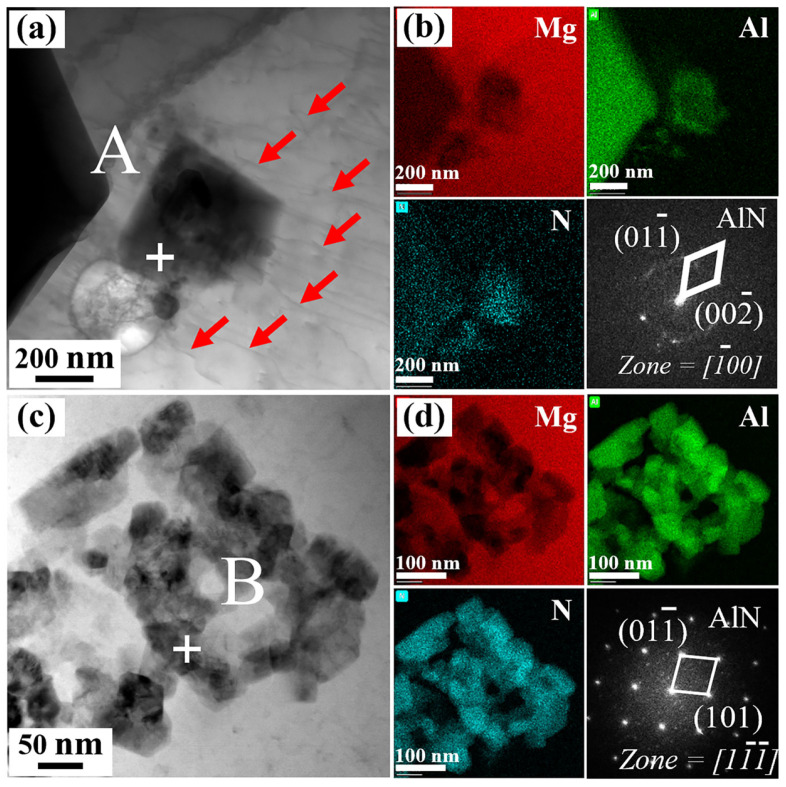
2.0 wt.% AlN/AE42 composites: (**a**) TEM micrograph of AlN particles, (**b**) EDS mapping and SAED micrograph of point A; 3.0 wt.% AlN/Mg-4Al-2Nd-0.3Mn composites: (**c**) TEM micrograph of AlN particles, (**d**) EDS mapping and SAED micrograph of point B.

Figure 8 presents TEM characterization of AlN particles in 2.0 wt.% and 3.0 wt.% AlN/AE42 composite. Nanometer AlN particles exist independently, with a size of approximately 512 nm, and there are many dislocations around the AlN particles, as indicated by the red arrows (Figure 8a). In contrast, AlN particles in the 3.0 wt.% composite (Figure 8c,d) exhibit significant agglomeration, and the size of the agglomeration area is approximately 1192 nm in diameter. This particle agglomeration reduced heterogeneous nucleation sites for α-Mg grains, resulting in coarsened α-Mg grains (Figure 2d) that diminished grain refinement strengthening. Furthermore, particle-enriched regions readily induced stress concentration [26], severely compromising AlN/α-Mg interface bonding strength.

### 3.2. Mechanical Properties

#### 3.2.1. Room-Temperature Mechanical Properties

**Figure 9:**
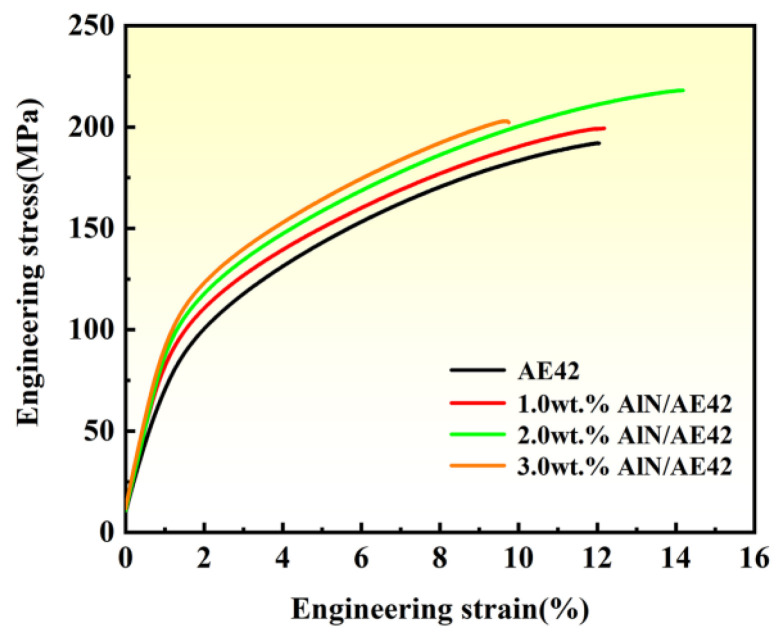
Tensile stress—strain curves of AlN/AE42 composites with different AlN contents at room temperature.

Room-temperature tensile properties of the AE42 alloy and composites are presented in Figure 9, with statistical results listed in Table 1. AE42 alloy exhibited YS, UTS, ε of 86 MPa, 191 MPa, and 12.1%, respectively. Composites with 1.0 wt.% and 2.0 wt.% AlN showed simultaneous enhancement in both strength and plasticity, with improvement magnitudes increasing alongside AlN content. The 2.0 wt.% AlN/AE42 composite achieved peak UTS (219 MPa) and ε (13.8%), with a YS of 107 MPa. Compared to the AE42 alloy, this represented increases of 21 MPa in YS, 28 MPa in UTS, and 1.7% in ε. At 3.0 wt.% AlN, YS further rose to 111 MPa, while UTS (203 MPa) and ε (9.8%) decreased slightly. Comparative analysis of SEM images (Figure 2) and grain sizes (Figure 4) revealed that AlN addition not only optimized the size and distribution of Al_2_Nd and Al_11_Nd_3_ phases but also refined α-Mg grains. This synergistic effect enhanced mechanical properties, granting the 2.0 wt.% AlN/AE42 composite superior overall performance.

**Figure 10:**
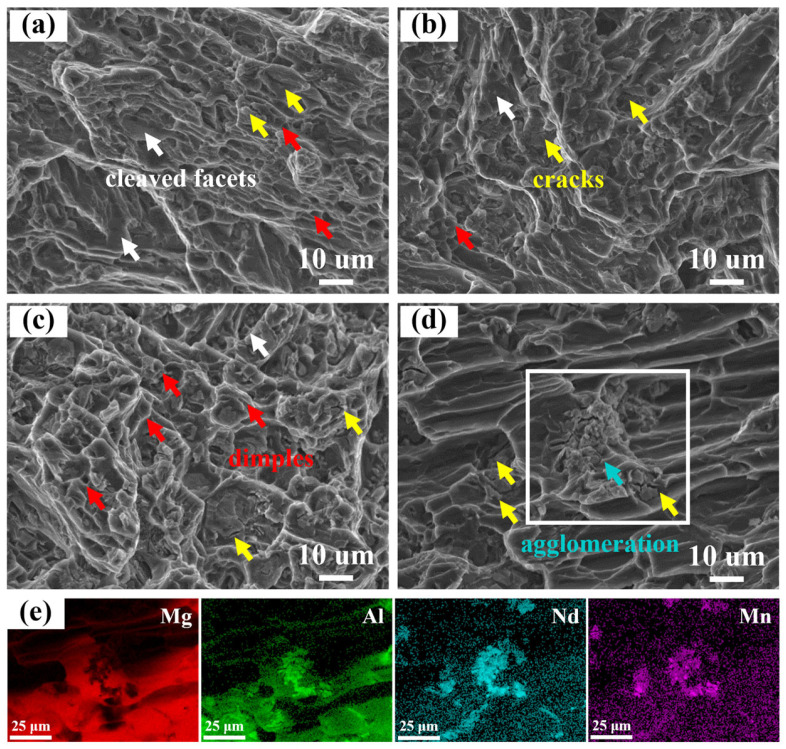
Tensile fracture morphology of AlN/AE42 composites at room temperature: (**a**) AE42, (**b**) 1.0 wt.% of AlN/AE42, (**c**) 2.0 wt.% of AlN/AE42, (**d**) 3.0 wt.% of AlN/AE42, (**e**) the EDS mappings of (**d**).

Fracture surfaces of AE42 alloy and composites at room temperature are shown in Figure 10. It was observed that there was a large area of cleavage plane on the fracture surface of AE42 alloy (as indicated by the white arrows in Figure 10a). In 1.0 and 2.0 wt.% AlN/AE42 composites, cleavage planes progressively diminished while tear ridges became increasingly tortuous with higher AlN content, indicating enhanced plasticity. In addition, second-phase agglomeration was observed in the fracture of 3.0 wt.% AlN/AE42 composites. The EDS elemental mapping analysis indicated that the agglomerate was composed of Al_x_Nd_y_ phase and Al-Mn phase (Figure 10e). In the 3.0 wt.% AlN/AE42 composites, due to the stress concentration caused around the agglomerated Al_x_Nd_y_ phase and Al-Mn phase, microcracks initiated and grew, eventually leading to the premature fracture of the composites and a decrease in plasticity [27].

#### 3.2.2. High-Temperature Mechanical Properties

**Figure 11:**
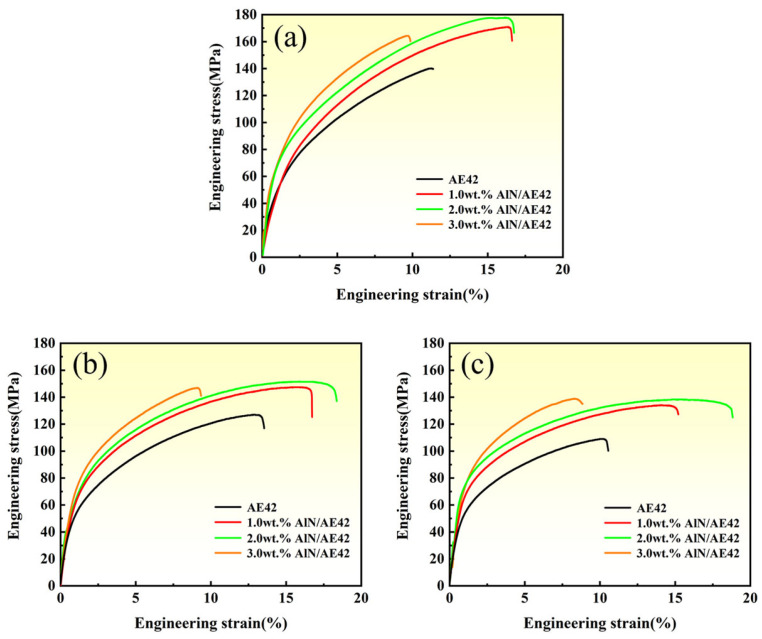
Tensile stress–strain curves of extruded alloys at the following high temperatures: (**a**) 150 °C, (**b**) 200 °C, and (**c**) 250 °C.

The high-temperature tensile properties of AE42 alloy and its composites at 150 °C, 200 °C, and 250 °C are presented in Figure 11, with corresponding data summarized in Table 2. The composites exhibited superior mechanical properties to the base alloy, where UTS and ε initially increased then decreased with rising AlN content, while YS progressively increased. The comprehensive performance of the 2.0 wt.% AlN/AE42 composite reached the optimal level. When tensile tests were performed at 150 °C, the 2.0 wt.% AlN/AE42 composite’s YS, UTS, and ε reached 84 MPa, 178 MPa, and 16.7%, respectively, representing enhancements of 14 MPa, 38 MPa, and 5.3% over AE42 alloy (70 MPa, 140 MPa, 11.4%). When tensile tests were performed at 200 °C, corresponding values reached 74 MPa, 152 MPa, and 18.3%, exceeding the AE42 alloy (62 MPa, 127 MPa, 13.0%) by 12 MPa, 25 MPa, and 5.3%. When tensile tests were performed at 250 °C, corresponding values reached 77 MPa, 139 MPa, and 18.8%, surpassing the AE42 alloy (54 MPa, 109 MPa, 10.4%) by 23 MPa, 30 MPa, and 8.4%. Furthermore, compared with the results of room temperature tensile tests, the UTS of the 2.0 wt.% AlN/AE42 composite decreased by 18.7% at 150 °C, while the ε increased by 2.9%. The UTS of the AE42 alloy decreased by 26.7% and the ε also decreased by 0.7%. This phenomenon confirmed that nano-AlN particles enhanced the comprehensive high-temperature mechanical properties of AE42 alloy while mitigating its performance degradation at high temperatures.

**Figure 12:**
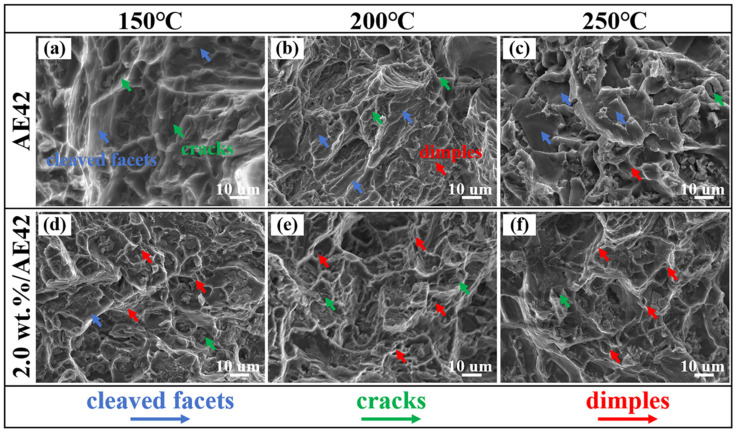
Tensile fracture morphology of AlN/AE42 composites at high temperature: (**a**–**c**) AE42, (**d**–**f**) 2.0 wt.% AlN/AE42.

Figure 12a–c demonstrate that the fracture surface of AE42 alloy after 150 °C tensile testing featured extensive cleavage planes with minor microcracks, indicating predominantly brittle fracture. At 200 °C, large cleavage planes diminished in the fracture surfaces while the number of dimples increased, accompanied by a certain number of microcracks, corresponding to improved plasticity at 200 °C. The tensile fracture surface at 250 °C was predominantly composed of extensive cleavage planes accompanied by a small number of large, shallow dimples. Simultaneously, numerous second-phase agglomerations were observed at the fracture surface, which might be the reason for the decrease in plasticity when tensile tests were performed at 250 °C. For the 2.0 wt.% AlN/AE42 composite (Figure 12d–f), fracture surfaces exhibited significantly reduced cleavage areas and higher densities of finer dimples compared to AE42 alloy. Additionally, there are more blocky second-phase particles that were fragmented within the dimples. With increasing tensile temperature, the dimples became deeper and more numerous, indicating significantly improved plasticity in the 2.0 wt.% AlN/AE42 composite, and the fracture mode was a mixed ductile-brittle fracture. The uniformly dispersed granular second phases hindered the propagation of cracks [28,29], reducing the rate of crack propagation and thereby enhancing the plasticity of the composite.

**Figure 13:**
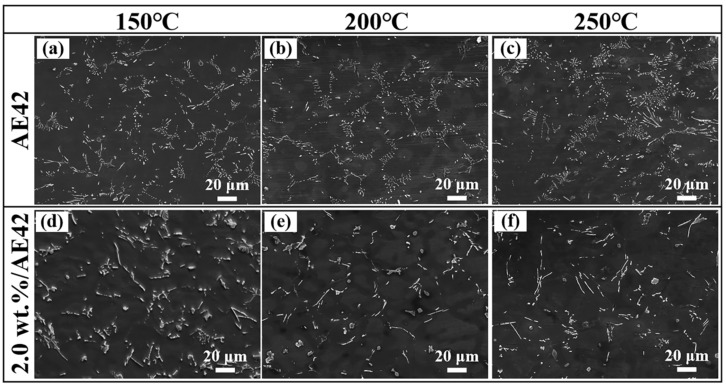
Microstructure characterization of AlN/AE42 composites after tensile testing at high temperature: (**a**–**c**) AE42, (**d**–**f**) 2.0 wt.% AlN/AE42.

Microstructural characterization near high-temperature fracture surfaces along the tensile direction is presented in Figure 13. In the AE42 alloy, a petal-like structure composed of Al_2_Nd and Al_11_Nd_3_ phases could still be observed after tensile testing at 150 °C. After tensile testing at 200 °C, the distribution of the second phase in the petal-like structure became denser (Figure 13b). After tensile testing at 250 °C, the petal-like structure in the AE42 alloy decreased, and the second phase exhibited agglomeration (Figure 13c). The large-scale agglomeration of the second phase was also one of the reasons for the failure of the AE42 alloy. At the same temperature, in the 2.0 wt.% AlN/AE42 composite, the distribution of the Al_11_Nd_3_ phase in the petal-like structure significantly improved, and the morphology of the Al_2_Nd phase also changed. The number of blocky Al_2_Nd phases increased and grew into short rod-shaped phases (Figure 13d–f). Compared with the acicular Al_11_Nd_3_ phase in the petal-like structure, the uniformly dispersed short rod-shaped and acicular phases more effectively hindered the movement of dislocations and the propagation of cracks. Moreover, the short rod-shaped Al_2_Nd phase exhibited morphological advantages in impeding dislocation movement, which more effectively enhanced the mechanical properties of the composite [30,31].

## 4. Discussion

### 4.1. Room-Temperature Strengthening Mechanism

Grain refinement strengthening and Orowan strengthening were the main strengthening mechanisms of AlN/AE42 composites at room temperature. The size of the grains had a significant impact on the mechanical properties of Mg alloys. According to the Hall–Petch relationship [32], the relationship between grain refinement strengthening (*σ_gs_*) and the average grain size (*d*) could be expressed as the following:

(1) $$\sigma_{gs}={kd}^{-1/2}$$

In the formula where *k* was constant, the finer the grain size, the more significant the grain refinement strengthening. The AE42 alloy, 1.0 wt.%, 2.0 wt.%, and 3.0 wt.% AlN/AE42 composites exhibited average grain sizes of 116.0 μm, 53.2 μm, 36.0 μm, and 50.6 μm, respectively. The 2.0 wt.% AlN/AE42 composite demonstrated the smallest grain size, representing a 70.0% reduction compared to AE42 alloy. This indicated that there were more grain boundaries to hinder the movement of dislocations, and the applied stress could be uniformly distributed to more grains, reducing stress concentration, which helped improve the strength and plasticity of the composite [33].

Orowan strengthening was one of the most significant strengthening mechanisms in Mg matrix composites. The nano-AlN particles prepared through an in situ reaction had good interface bonding with α-Mg. Introducing these in situ AlN particles into the Mg melt through the master alloy + casting route achieved uniform distribution within AlN/AE42 composites while preventing particle surface contamination. The uniformly dispersed nano-AlN particles in the matrix acted as a difficult-to-shear reinforcement phase during tensile deformation, effectively hindering the movement of dislocations, thereby significantly improving the tensile properties of the composite [34]. Furthermore, the addition of AlN particles reduced the size of the second phase and improved its distribution, as demonstrated in Figure 4. The reduction in the size of the Al_11_Nd_3_ and Al_2_Nd phase meant that the composite contained a greater number of fine Al_x_Nd_y_ phases, which contributed to improved composite YS. At the same time, numerous Al_8_Mn_5_ nanoparticles in the 2.0 wt.% AlN/AE42 composite also hindered the movement of dislocations (Figure 6d), effectively enhancing mechanical performance.

However, the 3.0 wt.% AlN/AE42 composite exhibited simultaneous reductions in both fracture strain and strength. This was attributed to the fact that as the AlN content increased, the content of Al introduced into the composite also increased. Excessive Al elements formed large-sized Al_6_Mn phases and grew interdependently with Al_x_Nd_y_ phases to form agglomerated areas. (Figure 7a). During tensile deformation, stress concentration was more likely to occur at the agglomeration areas of the second phase, leading to the formation of crack sources and subsequently causing the failure of the composite, as shown in Figure 10d.

### 4.2. High-Temperature Strengthening Mechanism

**Figure 14:**
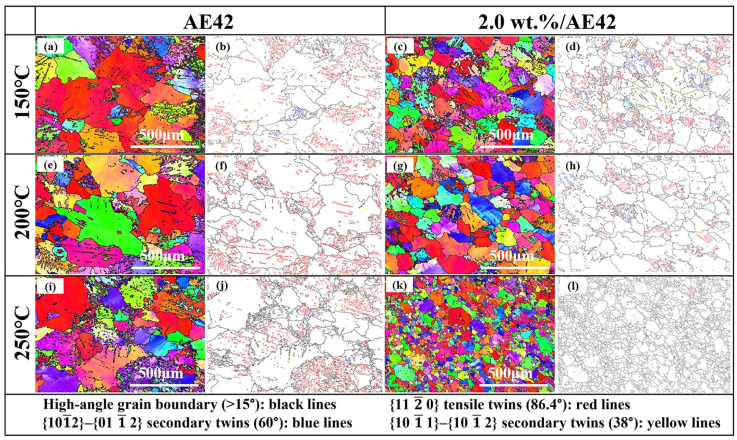
EBSD images of the AE42 alloy after high-temperature tensile tests: (**a**,**b**) 150 °C, (**e**,**f**) 200 °C, (**i**,**j**) 250 °C; EBSD images of 2.0 wt.% AlN/AE42 composites after high-temperature tensile tests: (**c**,**d**) 150 °C, (**g**,**h**) 200 °C, (**k**,**l**) 250 °C.

To explore the strengthening mechanism of the AlN/AE42 composite at high temperatures, EBSD characterization was carried out on the microstructure near the fracture surface parallel to the tensile deformation direction (Figure 1b). The result is shown in Figure 14. After high-temperature tensile testing at 150 °C and 200 °C, obvious banded structures were observed in both the AE42 alloy and the 2.0 wt.% AlN/AE42 composite. Due to the lack of sufficient slip systems during deformation, AE42 alloy formed a small amount of twins to coordinate deformation, thereby maintaining good plasticity at high temperatures. The twinning content in the 2.0 wt.% AlN/AE42 composite was significantly higher than that in AE42 alloy. The twinned contents in AE42 alloy and the 2.0 wt.% AlN/AE42 composite after tensile testing at 150 °C were 9.6% and 14.4%, respectively, while at 200 °C they were 8.9% and 15.4%, respectively. Only {10$\bar{1}$2} tensile twins were activated within AE42 alloy grains. In the 2.0 wt.% AlN/AE42 composite, there existed not only abundant {10$\bar{1}$2} tensile twins but also numerous {10$\bar{1}$2}–{01$\bar{1}$2} (60°) secondary twins and limited {10$\bar{1}$1}–{10$\bar{1}$2} (38°) secondary twins. The {10$\bar{1}$1}–{10$\bar{1}$2} twins required higher CRSS than {10$\bar{1}$2} twins, thus demanding greater stress concentration for activation. This indicated that AlN particles promoted more diverse and numerous twins by creating additional stress concentration sites, increasing overall twin content. These twins enhanced plasticity by releasing stress concentration and coordinating plastic deformation; on the other hand, twin boundaries reduced α-Mg grain size and acted as barriers to dislocation motion, increasing strain hardening rates [35,36]. Simultaneously, the refined α-Mg grains and Al_x_Nd_y_ phases also exerted a more significant inhibitory effect on dislocations, enabling the composite to exhibit superior comprehensive mechanical performance during high-temperature tensile tests.

After tensile testing at 250 °C, the twin content of AE42 alloy was 7.3%, and that of 2.0 wt.% AlN/AE42 composite was 2.2%. In the 2.0 wt.% AlN/AE42 composite, the twinned content was relatively low, and a distinct dynamic recrystallization (DRX) phenomenon occurred (Figure 14k), with the DRXed grains averaging a size of approximately 22.3 μm. This was due to the strain mismatch between the hard AlN particles, the fine Al_x_Nd_y_ phase, and the ductile matrix during the high-temperature tensile deformation process [37,38]. This mismatch led to the generation of a strain gradient in the matrix near the AlN particles and the Al_x_Nd_y_ second phase, resulting in a higher dislocation density in the region near the phase interface. This provided a greater driving force for DRX, thereby generating DRX and enhancing the strength and fracture strain of the 2.0 wt.% AlN/AE42 composite after high-temperature deformation at 250 °C. In addition, the nano-AlN particles could also pin grain boundaries, enhancing the stability of grain boundaries, hindering their migration, and strengthening the inhibitory effect of grain boundaries on dislocations, thereby promoting DRX at the grain boundaries while restricting the growth of DRXed grains. Therefore, on the one hand, the AlN particles could promote the proliferation of dislocations, thereby facilitating the nucleation of DRXed grains; on the other hand, the pinning effect of AlN particles on dislocations and grain boundaries effectively hindered grain growth, which was conducive to the improvement of high-temperature mechanical properties [39]. Thanks to the strengthening effect of AlN particles, the 2.0 wt.% AlN/AE42 composite had the best comprehensive mechanical properties after undergoing high-temperature deformation at 250 °C.

**Figure 15:**
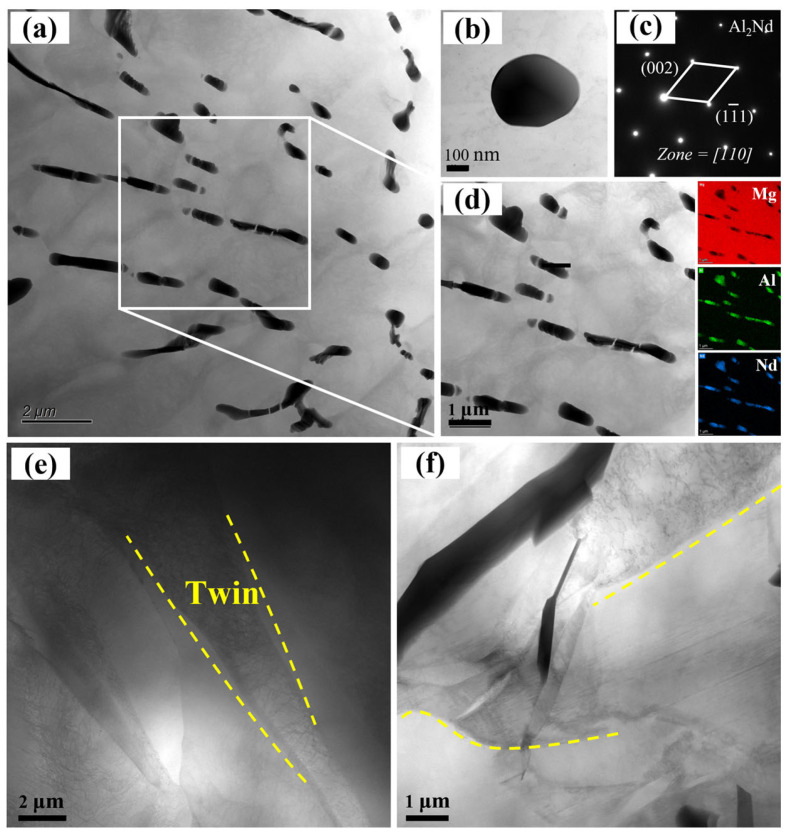
TEM micrograph of the AE42 alloy and 2.0 wt.% AlN/AE42 composite after tensile testing at 250 °C: (**a**) morphology change in the needle second phase in the AE42 alloy, (**b**) Al_2_Nd particles, (**c**) diagram of SAED, (**d**) composition identification of the second phase, (**e**) Twins in the AE42, (**f**) twins in the 2.0 wt.% AlN/AE42.

The TEM image of AE42 alloy after high-temperature tensile testing at 250 °C is shown in Figure 15a–d. A small amount of the Al_11_Nd_3_ phase was observed to decompose into Al_2_Nd phase in the AE42 alloy. The decomposition of the Al_11_Nd_3_ second phase at high temperatures made it unable to effectively impede the movement of grain boundaries and dislocations, thereby significantly reducing the mechanical properties of AE42 alloy. Figure 15e and Figure 15f, respectively, show the typical morphologies of twins in AE42 alloy and 2.0 wt.% AlN/AE42 composite after tensile testing at 250 °C. In the AE42 alloy, the twin interfaces were very sharp and clear, and the dislocation density within the twins was very high. In the 2.0 wt.% AlN/AE42 composite, the twin interfaces became more blurred, the twins were discontinuous and incomplete, and the dislocation density within the twins decreased. This indicated that DRX had occurred in the composite, and the twins were consumed by DRX. Wang et al. [40] also found that after compression at 250 °C, the dislocation density was high and the entanglement was severe; meanwhile, after compression at 350 °C, due to DRX, the dislocation density was significantly reduced. In this study, the large-area DRX in the 2.0 wt.% AlN/AE42 composite enabled it to exhibit better comprehensive mechanical properties when tensile testing was performed at 250 °C.

## 5. Conclusions

(1) With increasing AlN content, the average sizes of the α-Mg grains and acicular (Al_11_Nd_3_) and blocky (Al_2_Nd) second phases in the AE42 alloy first decreased and then increased while promoting the precipitation of the Al8Mn5 phase. Optimal refinement effects were achieved in the 2.0 wt.% AlN/AE42 composite, where average sizes of α-Mg, Al_11_Nd_3_, and Al_2_Nd phases in AE42 alloy were reduced from 116.0 μm, 7.9 μm, and 4.3 μm to 36.0 μm, 5.7 μm and 2.6 μm, respectively.
(2) Benefiting from the refinement effects of nano-AlN particles on α-Mg grains and Al_x_Nd_y_ phases, coupled with their dislocation pinning effect, the 2.0 wt.% AlN/AE42 composite achieved enhanced room-temperature properties: YS, UTS, and ε reached 107 MPa, 219 MPa, and 13.8%, respectively.
(3) The addition of nano-AlN particles promoted twin precipitation and DRX in AE42 alloy during high-temperature tensile deformation. Furthermore, the grain boundary pinning effect of AlN particles hindered the growth of DRXed grains while simultaneously enhancing grain boundary stability, effectively improving the high-temperature mechanical properties of AlN/AE42 composites. In the 2.0 wt.% AlN/AE42 composite, YS, UTS, and ε at 250 °C were 77 MPa, 139 MPa, and 18.8%, respectively, representing increases of 23 MPa, 30 MPa, and 8% over the AE42 alloy (54 MPa, 109 MPa, 10.8%).

## Figures and Tables

**Table 1:** Tensile properties of AlN/AE42 composites at room temperature.

Samples	YS/MPa	UTS/MPa	ε/%
AE42	86 ± 1	191 ± 2	12.1 ± 0.2
1.0 wt.% AlN/AE42	100 ± 2	199 ± 2	13.2 ± 0.2
2.0 wt.% AlN/AE42	107 ± 2	219 ± 1	13.8 ± 0.3
3.0 wt.% AlN/AE42	111 ± 4	203 ± 3	9.8 ± 0.3

**Table 2:** Tensile properties of extruded alloys at high temperatures.

Temperature	Alloys	YS (MPa)	UTS (MPa)	ε (%)
150 °C	AE42	70 ± 3	140 ± 2	11.4 ± 0.3
	1.0 wt.% AlN/AE42	81 ± 3	171 ± 3	16.5 ± 0.4
	2.0 wt.% AlN/AE42	84 ± 2	178 ± 3	16.7 ± 0.3
	3.0 wt.% AlN/AE42	90 ± 3	164 ± 4	9.8 ± 0.5
200 °C	AE42	62 ± 3	127 ± 3	13.0 ± 0.4
	1.0 wt.% AlN/AE42	71 ± 2	147 ± 3	16.1 ± 0.3
	2.0 wt.% AlN/AE42	74 ± 2	152 ± 3	18.3 ± 0.4
	3.0 wt.% AlN/AE42	83 ± 4	147 ± 5	9.3 ± 0.5
250 °C	AE42	54 ± 3	109 ± 2	10.8 ± 0.2
	1.0 wt.% AlN/AE42	71 ± 2	134 ± 3	15.1 ± 0.4
	2.0 wt.% AlN/AE42	77 ± 3	139 ± 2	18.8 ± 0.3
	3.0 wt.% AlN/AE42	79 ± 5	139 ± 3	8.7 ± 0.5

## Data Availability

The original contributions presented in this study are included in the article. Further inquiries can be directed to the corresponding authors.
